# Enhancing electric-field control of ferromagnetism through nanoscale engineering of high-*T*_c_ Mn_*x*_Ge_1−*x*_ nanomesh

**DOI:** 10.1038/ncomms12866

**Published:** 2016-10-20

**Authors:** Tianxiao Nie, Jianshi Tang, Xufeng Kou, Yin Gen, Shengwei Lee, Xiaodan Zhu, Qinglin He, Li-Te Chang, Koichi Murata, Yabin Fan, Kang L. Wang

**Affiliations:** 1 Department of Electrical Engineering, Device Research Laboratory, University of California, Los Angeles, California 90095, USA; 2 Institute of Materials Science and Engineering, National Central University, 300 Jung-Da Rd, Chung-Li 320, Taiwan

## Abstract

Voltage control of magnetism in ferromagnetic semiconductor has emerged as an appealing solution to significantly reduce the power dissipation and variability beyond current CMOS technology. However, it has been proven to be very challenging to achieve a candidate with high Curie temperature (*T*_c_), controllable ferromagnetism and easy integration with current Si technology. Here we report the effective electric-field control of both ferromagnetism and magnetoresistance in unique Mn_*x*_Ge_1−*x*_ nanomeshes fabricated by nanosphere lithography, in which a *T*_c_ above 400 K is demonstrated as a result of size/quantum confinement. Furthermore, by adjusting Mn doping concentration, extremely giant magnetoresistance is realized from ∼8,000% at 30 K to 75% at 300 K at 4 T, which arises from a geometrically enhanced magnetoresistance effect of the unique mesh structure. Our results may provide a paradigm for fundamentally understanding the high *T*_c_ in ferromagnetic semiconductor nanostructure and realizing electric-field control of magnetoresistance for future spintronic applications.

## Abstract (web summary)

Voltage control of magnetism in ferromagnetic semiconductor is appealing for spintronic applications, which is yet hindered by compound formation and low Curie temperature. Here, Nie *et al*. report electric-field control of ferromagnetism in Mn_x_Ge_1−x_ nanomeshes with a Curie temperature above 400 K and controllable giant magnetoresistance.

## Graphical abstract

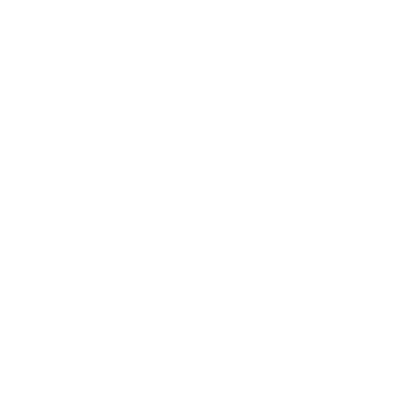

For low power dissipation and low variability in the continuous scaling of complementary metal-oxide-semiconductor (CMOS), the search for novel materials and devices has been highlighted in the International Technology Roadmap of Semiconductors^1^,^2^. Spintronics, which utilizes the spin of electrons as another d.f. for information processing, offers a promising pathway to meet this challenge^3^. Recently, the use of spin-polarized current to manipulate magnetism^2^,^4^ has drawn great attentions for low-power spintronics devices. Further significant reduction of switching power can be envisaged by using an electric field rather than current. With carrier-mediated ferromagnetism, ferromagnetic semiconductors (FMS)^5^,^6^ can open up great opportunities in realizing voltage controlled non-volatile and low-power spintronic devices, such as magnetoresistance (MR)-type memories^7^, magnetic-field sensors^8^ and spinFETs^9^,^10^. Among various FMS materials, Mn_*x*_Ge_1−*x*_ has attracted significant attentions due to its compatibility with the mature Si technology and its potentially high Curie temperature (*T*_c_)^11^,^12^. In practice, however, the realization of high-quality and high-*T*_c_ Mn_*x*_Ge_1−*x*_ thin films with electric-field-controlled ferromagnetism has been proven to be difficult due to the extremely low Mn solubility in Ge. Intermetallic compounds (such as Mn_5_Ge_3_ and Mn_11_Ge_8_) and Mn-rich nanophases^8^ are usually formed in a random and uncontrollable manner^12^,^13^,^14^. Although great efforts have been devoted to avoid the formation of these compounds, completely eliminating them remains elusive. Therefore, the quest for spintronic devices to work at ambient temperature calls for a new material candidate or structure, in which a high *T*_c_ can be attained with controllable ferromagnetism and easy integration with current CMOS platform.

For seeking such new candidates, the pioneer work on one-dimensional (1D) Mn_*x*_Ge_1−*x*_ nanowires^15^,^16^ grown by chemical vapour deposition has shown a *T*_c_ over 300 K, however, the electric-field control of ferromagnetism was never demonstrated^16^,^17^. Later, our molecular beam epitaxy (MBE)-grown Mn_*x*_Ge_1−*x*_ quantum dots (QDs)^11^ were also found to exhibit a *T*_c_ higher than room temperature, and more significantly we demonstrated electric-field controlled ferromagnetism. The underlying mechanism of the high *T*_c_ was attributed to the strong quantum confinement effect, which significantly enhanced the exchange coupling between the confined holes and localized Mn ions^11^,^17^,^18^,^19^. Those inspiring results point to a new direction of using nanostructures to realize high-*T*_c_ Mn_*x*_Ge_1−*x*_ FMS. However, Mn_*x*_Ge_1−*x*_ QDs suffer from high growth temperature, inhomogeneity from self-assembly growth and inherent difficulty for transistor. And above all, it is difficult to process them for practical devices. Likewise, for 1D nanowires, the fabrication process usually relies on low-throughput e-beam lithography^20^. To overcome the above obstacles, new nanostructures must be produced in a large-scale uniform fashion with easy device fabrication and convenient integration with current Si technology. Nanomesh^21^ appears to be an attractive candidate since it can be easily fabricated in large scale with high throughput and good uniformity.

Commonly, two strategies could be employed to realize nanomesh structures^21^,^22^. One is post-synthesis top-down etching the two-dimensional film into nanomesh structures^23^, and the other is to form a nanopattern first followed by the growth of nanomesh structures^24^. In comparison, the former method still suffers the aforementioned precipitates problem, and unavoidably produces rough and disordered edges during the etching process, which may also give rise to additional carriers scattering and reduce carrier mobility^22^. Therefore, the latter one is preferred for the high-quality nanomesh fabrication. Among nanofabrication methods, nanosphere lithography is a good choice due to its simplicity, low cost and high throughput in the fabrication of large-area and highly-ordered patterns^25^. Furthermore, the pattern size can be easily controlled by choosing specific nanosphere diameters and by adjusting the etching time. All these advantages make it extremely suitable for nanomesh fabrication in large scale with a controllable size.

Herein, wafer-scale Mn_*x*_Ge_1−*x*_ nanomeshes with controlled Mn-doping concentrations are successfully grown by MBE at low temperature on a pre-patterned Ge substrate fabricated by nanosphere lithography. The nanomesh width can be well controlled by oxygen-plasma etching of the nanospheres. Comprehensive transmission electron microscopy (TEM) characterizations of the nanomeshes clearly show the coherent growth of the Mn_*x*_Ge_1−*x*_ nanomesh on Ge substrate, without observable intermetallic precipitates. The *T*_c_ of the Mn_*x*_Ge_1−*x*_ nanomesh is found to be above 400 K measured by a superconducting quantum interference device (SQUID). Furthermore, the magnetotransport measurement demonstrates the MR can be controlled from positive and negative values by applying a gate bias. With an appropriate Mn doping concentration, a giant MR as high as 8,000% is obtained at low temperature. We attribute the giant MR to a geometrical enhancement of the unique nanomesh structure. The successful growth and engineering of the high-*T*_c_ Mn_*x*_Ge_1−*x*_ nanomesh with a tunable giant and electric-field controlled MR have the potential to exploit the Ge-based spintronics devices, such as spin field-effect transistor (spinFET)^9^, magnetic random-access memory^26^,^27^ and magnetic-field sensor^8^, among others.

## Results

### Pattern design

**Figure 1:**
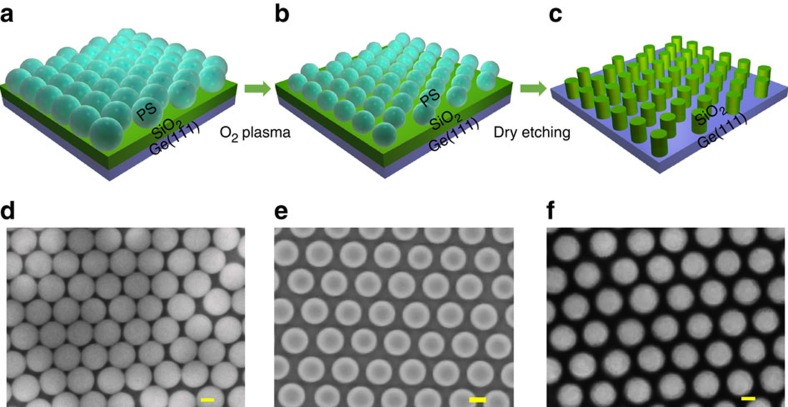
Process flow diagrams for the pattern fabrication. (**a**) Self-assembly growth of closely-packed single layer of nanospheres on the Ge substrate. (**b**) O_2_-plasma etching of the nanospheres to reach the desired size. (**c**) SiO_2_ nanopillars formed by dry etching, masked by the nanospheres. (**d**–**f**) The corresponding SEM images of every steps. Scale bar, 100 nm.

Figure 1a–c shows the process-flow diagrams on how to fabricate periodic pattern on Ge substrate. After self-assembled patterning nanospheres, a typical scanning electron microscopy (SEM) image (see Fig. 1d) clearly shows the close-packed single layer of nanospheres with a diameter of 220 nm. By controlling the O_2_ plasma-etching rate, the nanospheres diameter is successfully shrunk to 160 nm with a 60 nm gap between them as shown in Fig. 1e. The nanosphere pattern was then transferred onto the SiO_2_ layer by dry etching firstly, followed with short-time wet etching. Due to the anisotropic dry etching, SiO_2_ nanopillars were obtained, between which the Ge substrate was exposed for the nanomesh growth. The periodic SiO_2_ nanopillars are clearly shown in the SEM image (Fig. 1f).

### Structural characterization of the Mn_*x*_Ge_1−*x*_ nanomesh

**Figure 2:**
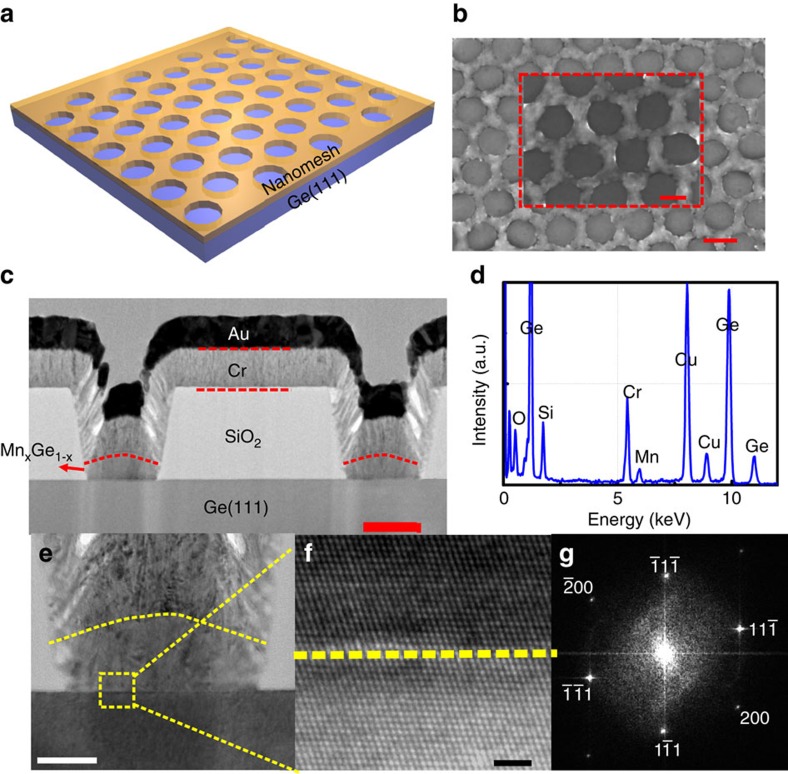
Structural properties of Mn_*x*_Ge_1−*x*_ nanomeshes. (**a**) Schematic illustration of the formed Mn_*x*_Ge_1−*x*_ nanomeshes. (**b**) Typical SEM image of the Mn_*x*_Ge_1−*x*_ nanomeshes, with a nanomesh width of 60 nm and a nanohole diameter of 160 nm. Scale bar, 200 nm. The inset is the magnified SEM image. Scale bar, 100 nm. (**c**) Typical cross-sectional TEM image of the Mn_*x*_Ge_1−*x*_ nanomeshes, in which the nanomeshes sit well on the Ge substrate and are spaced by the SiO_2_ mask. Scale bar, 50 nm. (**d**) EDS spectrum, showing a Mn composition of ∼3%. (**e**) Magnified cross-sectional TEM image of the Mn_*x*_Ge_1−*x*_ nanomesh. Scale bar, 20 nm. (**f**) HRTEM image of the interface between the Mn_*x*_Ge_1−*x*_ nanomesh and the Ge substrate, clearly showing that the Mn_*x*_Ge_1−*x*_ nanomesh is a single crystal. Scale bar, 2 nm. (**g**) Fourier-transform image of (**f**).

The SiO_2_ mask was subsequently removed after the MBE growth by selective etching and only the Mn_*x*_Ge_1−*x*_ nanomesh was left on the Ge substrate. Figure 2a is a schematic illustration of the Mn_*x*_Ge_1−*x*_ nanomesh, in which the yellow-colored nanomesh sits on top of the blue-colored Ge (111) substrate. The typical morphology of the sample was captured by SEM as shown in Fig. 2b. A periodic nanomesh structure has a nanomesh width of 60 nm and a hole diameter of 160 nm; a magnified SEM image is also shown in the inset. To further characterize the microstructure and composition of the formed nanomesh, the cross-sectional TEM was used and the results are shown in Fig. 2c–g, for which a focused ion beam was employed to cut the sample along the diameter of the hole. In this process, a Cr/Au layer was deposited to protect the sample from damage of the ion beam. Figure 2c is a low-resolution cross-sectional TEM image of the Mn_*x*_Ge_1−*x*_ nanomesh, which clearly shows that the nanomesh is grown on the Ge substrate as defined by the SiO_2_ pattern. The zoom-in image (Fig. 2e) shows that the Mn_*x*_Ge_1−*x*_ nanomesh has a height of ∼25 nm and a width of ∼60 nm, consistent with the SEM result. The high-resolution TEM (HRTEM) image (Fig. 2f) clearly demonstrates the coherent growth of the Mn_*x*_Ge_1−*x*_ nanomesh on Ge substrate. It does not reveal observable intermetallic compounds. The Fourier-transform image as shown in Fig. 2g gives only one set of periodic pattern, proving a perfectly epitaxial growth. This image can be indexed to the [011] zone axis of the Ge diamond lattice and the epitaxial growth direction is along [111]. To understand the nanomesh composition, energy-dispersive spectroscopy (EDS) was used and the result is given in Fig. 2d. The Mn doping concentration can be estimated to be ∼3%. The additional peaks, Cr and Si, come from the protection layer and the SiO_2_ pattern, respectively. The EDS mapping and line scan analysis can further confirm the uniform distribution of Mn in Mn_*x*_Ge_1−*x*_ nanomesh, shown in Supplementary Fig. 1. Combined with the HRTEM analysis, the grown Mn_*x*_Ge_1−*x*_ nanomesh is believed to be single crystalline FMS^11^.

### Magnetic properties of the Mn_*x*_Ge_1−*x*_ nanomesh

**Figure 3:**
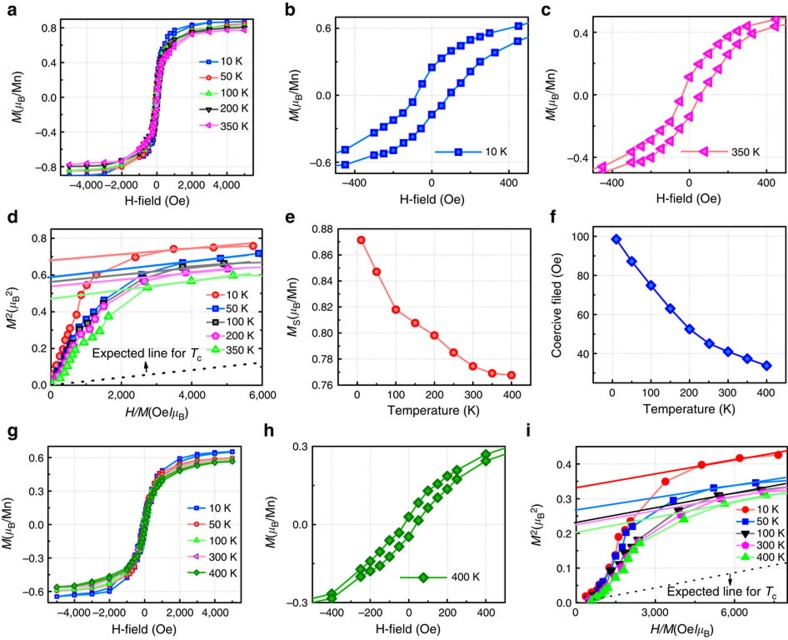
Magnetic properties of Mn_*x*_Ge_1−*x*_ nanomeshes. (**a**) Magnetic hysteresis loops of the Mn_0.03_Ge_0.97_ nanomeshes measured at different temperatures from 10–350 K. (**b**,**c**) The magnified hysteresis loop obtained at 10 and 350 K, respectively. (**d**) Arrott's plots showing that the *T*_c_ is above 350 K. (**e**) The temperature-dependent saturation moment. (**f**) Temperature-dependent coercivity decreasing from 100 Oe at 10 K to 35 Oe at 400 K. (**g**) Temperature-dependent *M*–*H* curves of the Mn_0.05_Ge_0.95_ nanomeshes. (**h**) The magnified hysteresis loop obtained at 400 K. (**i**) Arrott's plots showing that its *T*_c_ is above 400 K.

To well understand the magnetic property of the Mn_*x*_Ge_1−*x*_ nanomesh, comparative samples with different Mn doping concentrations (3% and 5%) are measured by SQUID. Figure 3a shows the temperature-dependent hysteresis loops of the 3% Mn-doped nanomesh (Mn_0.03_Ge_0.97_), when an external field is applied parallel to the sample surface. The S-shaped hysteresis loops indicate the ferromagnetism above 350 K^8^. Figure 3b is the magnified hysteresis loop obtained at 10 K, clearly showing a small coercivity of 100 Oe. The saturation magnetization (*M*_s_) per Mn is estimated to be 0.87 *μ*_B_ at 10 K. In addition, Arrott plots were also used to evaluate the *T*_c_, as shown in Fig. 3d. We observe that even at 350 K, the intercept, namely reciprocal of the susceptibility, does not vanish, indicating that the *T*_c_ has not been reached yet. The extrapolated dashed line further indicates the *T*_c_ is beyond 350 K, which agrees well with the hysteresis loops. Figure 3e shows the temperature-dependent *M*_s_ ranging from 10 to 400 K; it clearly shows a weak temperature dependence and a large magnetization remained at 400 K. All of the data support that the *T*_c_ is beyond 400 K. The temperature-dependent coercivity is shown in Fig. 3f, in which the coercivity decreases from 100 to 35 Oe with increasing temperature. The small coercivity in the entire investigated temperature range indicates that the soft ferromagnetism of our sample may come from the diluted nature of Mn^8^. The magnetic property of the 5% Mn-doped Mn_*x*_Ge_1−*x*_ nanomesh (Mn_0.05_Ge_0.95_) is shown in Fig. 3g–i. Figure 3g shows a similar temperature dependence of hysteresis loops as that in the Mn_0.03_Ge_0.97_ nanomesh. Furthermore, a high *T*_c_ (above 400 K) can also be seen from both the S-shaped hysteresis loop (Fig. 3h) and Arrott plot (Fig. 3i). However, a careful examination indicates that the saturated magnetic moment per Mn in the Mn_0.05_Ge_0.95_ nanomesh is a little smaller than that in the Mn_0.03_Ge_0.97_ nanomesh. The underlying mechanism may arise from the increased interstitial Mn density, as the Mn doping concentration increases^28^. It has been well documented that the Mn interstitials are ferromagnetically inactive^29^. Meanwhile, Mn interstitials acting as double donors^29^ can compensate a significant fraction of free holes, which can further weaken the hole-mediated ferromagnetism^6^,^30^.

To date, several papers have reported a similar high *T*_c_ phenomenon^8^,^14^,^15^,^16^,^31^ in Mn_*x*_Ge_1−*x*_ systems, which can be classified into two types of structures: Mn-rich Mn_*x*_Ge_1−*x*_ nanocolumn^8^ and Mn-diluted Mn_*x*_Ge_1−*x*_ nanostructure^14^,^15^,^16^,^31^. The Mn-rich Mn_*x*_Ge_1−*x*_ nanocolumn exhibited a *T*_c_ over 400 K^8^, but no electric-field control of ferromagnetism was reported. Recently, the nanocolumn has been proven to be new phase of MnGe_2_ (ref. 32). The Mn-diluted Mn_*x*_Ge_1−*x*_ nanostructures^14^,^15^,^16^,^31^, including nanowires and nanodots, usually displayed a *T*_c_ over 300 K. In particular, Mn_0.05_Ge_0.95_ QDs showed an electric-field controlled ferromagnetism. To explore the origin of the high *T*_c_ in our sample, the zero-field cooled (ZFC) and field cooled (FC) magnetization measurement were performed and the superimposed ZFC and FC curves (Supplementary Fig. 2) indicate there are no any nanophase precipitates^29^. Together with the TEM evidence, we believe the origin of the high *T*_c_ in our sample comes from the nanosize and quantum confinement effect of our Mn_*x*_Ge_1−*x*_ nanomesh. As the sample is scaled down to nanoscale, the quantum confinement effect in nanostructures can significantly boost the exchange coupling between the confined holes and the localized Mn^11^, leading to a higher *T*_c_ compared with bulk materials^1^,^11^,^17^. Additionally, when it comes to the nanostructure, the ability to accommodate the strain without formation of the lattice defects can be dramatically enhanced due to its large surface^33^,^34^. In the Mn_*x*_Ge_1−*x*_ thin film, the lattice strain associated with substitutional Mn can affect and limit the growth of high-quality FMS^35^; the Mn_*x*_Ge_1−*x*_ lattice swells up to a critical strain above which Mn atoms segregate into Mn-rich nanocrystals^35^. In the Mn_*x*_Ge_1−*x*_ nanomesh structure, however, the strain induced by Mn doping could be relieved due to the large surface area of the nanomesh and thus more Mn atoms can be accommodated into the substitutional position, giving rise to a higher *T*_c_ than thin films^29^,^36^,^37^. To understand the size confinement on the growth quality, we give a systematic ZFC and FC measurement on different size nanomesh structures, shown in Supplementary Fig. 2, and the result clearly proves the size confinement effect can suppress the compound formation and improve the *T*_c_ (detailedly discussed in Supplementary Note 1). Finally, we should point out that previously reported Mn-diluted nanostructures^14^,^15^,^16^,^31^, including the chemical vapour deposition-grown nanowires and self-assembly grown QDs, were usually formed at a high growth temperature. In comparison, our Mn_*x*_Ge_1−*x*_ nanomesh, which was grown at low temperature, can fully utilize the advantages of the non-equilibrium MBE technology. More Mn can be incorporated into the Ge lattice in our nanomesh structure in a non-equilibrium manner, and thus a much higher *T*_c_ can be expected. Overall, the unique use of Mn_*x*_Ge_1−*x*_ nanomeshes facilitates the formation of the high-*T*_c_ ferromagnetic semiconductor.

### Magnetotransport measurement of the Mn_*x*_Ge_1−*x*_ nanomesh

**Figure 4:**
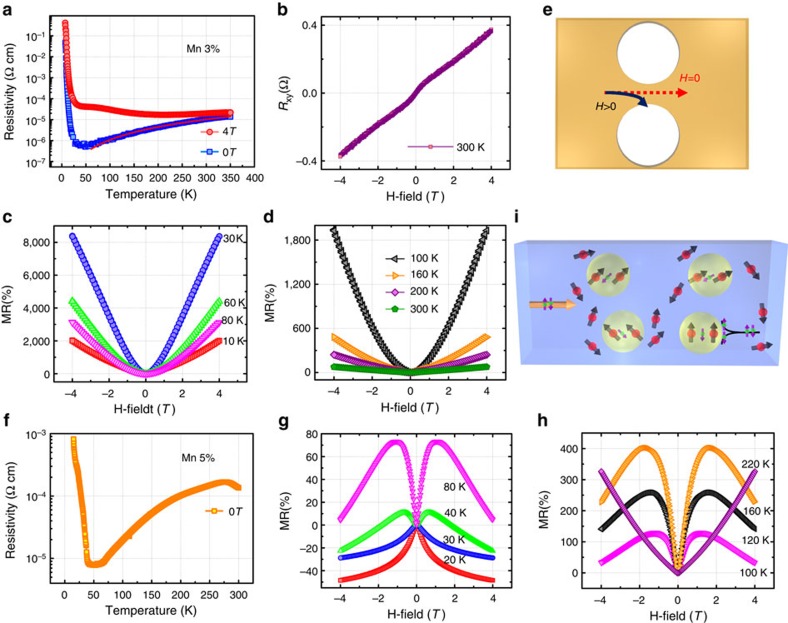
Temperature-dependent magnetotransport measurement of Mn_*x*_Ge_1−*x*_ nanomeshes. (**a**) Temperature-dependent resistivity of the Mn_0.03_Ge_0.97_ nanomeshes measured without an applied magnetic field (blue square) and with a magnetic field of 4 T (red circle), clearly showing a metal–insulator transition and a large resistivity deviation between them. The red solid line over the blue data is the fitting curve of a power-law relation to *T*^1.6^, indicating the carrier scattering by phonons. (**b**) The Hall resistance at 300 K. (**c**,**d**) Temperature-dependent MR measured at low-temperature and high-temperature regions, respectively. (**e**) The schematic illustration of scattering mechanism. (**f**) Temperature-dependent resistivity of the Mn_0.05_Ge_0.95_ nanomeshes, showing a much larger resistivity compared with that in the Mn_0.03_Ge_0.97_ nanomeshes. (**g**,**h**) Temperature-dependent MR measured at low-temperature and high-temperature regions, respectively, showing a MR transition from a negative to positive value. (**i**) The schematic illustration of two competitive scattering mechanisms from magnetic polarons in Mn_0.05_Ge_0.95_ nanomeshes.

Furthermore, the magnetotransport property of the Mn_0.03_Ge_0.97_ nanomesh was revealed by physical property measurement system (PPMS) and the results are shown in Fig. 4. Figure 4a shows the temperature-dependent resistivity of the Mn_0.03_Ge_0.97_ nanomesh under the magnetic field of 0 T (blue square) and 4 T (red circle), respectively, in which a huge difference can be observed. A clear metal-to-insulator transition without applying magnetic field (0 T) can be observed with a low-temperature (*T*<30 K) activation region and a high-temperature (*T*>30 K) saturation region. From the Arrhenius relation^38^, the activation energy of our Mn_0.03_Ge_0.97_ nanomesh is estimated to be ∼11 meV, which, however, is lower than the substitutional Mn acceptor energy level (∼160 meV)^11^. A similar low activation energy (30 meV) of Mn in Ge has also been reported in a previous study^39^. The underlying mechanism comes from the high-doping level and the presence of exchange interactions, inducing the broadening and possible splitting of the impurity band^39^. Above 30 K, the *R*–*T* curve could be well fitted by a power-law relation (*T*^*α*^) with *α*≈1.6 close to the value 1.5 predicted for hole scattering by phonons in Ge^8^. The fitting was plotted in red over the blue data in the *R*–*T* curve. As shown in Fig. 4b, the ordinary Hall coefficient at 300 K is positive, further proving that the majority carriers are holes provided by the substitutional Mn acceptors.

An intriguing phenomenon is the observation of a giant positive MR in the Mn_0.03_Ge_0.97_ nanomesh as shown in Fig. 4c,d, when an out-of-plane magnetic field was applied. At a magnetic field of 4 T, the MR as high as 2,000% at 10 K is observed and still 75% at 300 K; however, the maximum of 8,000% occurs at 30 K. Traditionally, the positive MR comes from the orbital MR, which is proportional to (*μB*)^2^, where *μ* is the carrier mobility^13^. However, the estimated orbital MR is too small to account for our giant MR. A similar MR effect has been reported previously in a Mn_*x*_Ge_1−*x*_ film, which was due to the formation of Mn-rich nanocolumns^8^,^14^. In our Mn_0.03_Ge_0.97_ nanomesh, the presence of such Mn-rich nanocolumns has been excluded from our extensive TEM analysis as discussed above. To reveal the reason behind the giant MR, an unpatterned Ge wafer was simultaneously loaded into the chamber for thin film growth as a reference, along with the Mn_0.03_Ge_0.97_ nanomesh sample. However, only ∼1% positive MR can be detected in the thin film (shown in Supplementary Fig. 3). Such distinct MR behaviours between the Mn_0.03_Ge_0.97_ nanomesh and thin film (see Supplementary Note 2) give the conclusion that the giant MR can only be attributed to the geometrically enhanced MR^26^,^40^ owing to the unique mesh structure. To understand it, the nanomesh structures can be considered as a highly conductive Mn_0.03_Ge_0.97_ percolation network with periodic nanoholes. Without applying a magnetic field, the current flows through the Mn_0.03_Ge_0.97_ nanomesh, and the current direction is parallel to the local electric field. As magnetic field is applied, the current is deflected due to the Lorentz force^41^; the current and the local electric field are no longer collinear. The angle between them is determined by the Hall angle *θ*=arctan(*μ*_H_*H*), where the *μ*_H_ is the Hall mobility. For a sufficiently large magnetic field, the current is obviously deflected from the highly conductive nanomesh to the insulated nanoholes, resulting in a high resistance. The transition from the extremely low resistance at zero magnetic field to the extremely high resistance at a large magnetic field gives rise to the giant MR for the Mn_0.03_Ge_0.97_ nanomesh, as illustrated in Fig. 4e. Thus, it may be concluded that the lower the initial resistance of the nanomesh is, the larger the MR became at a given magnetic field. It can be verified from the deviations of the *R*–*T* curves between 4 and 0 T, as already shown in Fig. 4a. The largest deviation happens at the lowest resistivity (at 30 K and 0 T), which agrees well with our proposed model.

For comparison, the magnetotransport property of the Mn_0.05_Ge_0.95_ nanomesh was also measured by PPMS, and the *R*–*T* curve is shown in Fig. 4f. A similar metal-to-insulator transition could be observed, however, the resistivity was found to be more than one order of magnitude larger than that of the Mn_0.03_Ge_0.97_ nanomesh. The increased resistivity may come from the increased scattering centres from the higher Mn doping concentration^42^. Figure 4g,h shows the temperature-dependent MR of the Mn_0.05_Ge_0.95_ nanomesh sample. Intriguingly, the geometrically enhanced giant MR becomes less pronounced in the Mn_0.05_Ge_0.95_ nanomesh sample. This may be due to the dramatically increased resistivity and the decreased Hall angle from the lower mobility compared with that in the Mn_0.03_Ge_0.97_ nanomesh. In addition, the Mn_0.05_Ge_0.95_ nanomesh shows a negative MR for temperature below 40 K and a positive MR above 160 K. In the intermediate temperature region (40–160 K), the MR contains two contributions: a positive MR appears at low magnetic field, followed by a negative slope at high field. From previous reports^29^,^43^, such negative-to-positive MR transition could be attributed to two competitive effects: the spin-dependent scattering by ferromagnetic clusters^29^,^44^ or magnetic polarons^29^,^44^ gave rise to the negative MR, while their spatial fluctuations led to the positive MR^29^,^44^. Since the presence of ferromagnetic clusters in our nanomesh sample has been excluded from the above detailed microstructural analysis and ZFC and FC measurement, the two competitive effects (illustrated in Fig. 4i) most likely come from the magnetic polarons 44,45,46, which are the origin of the ferromagnetism in the FMS. The radius of the polaron, *r*_h_, grows with decreasing temperature defined by *r*_h_∼(*a*_b_/2)ln(*sS*|*J*_0_|/(*k*_B_*T*)), where *a*_b_ is the decay length of the localized carrier, *s* and *S* are the absolute values of the hole and impurity spin, respectively and *J*_0_ is the strength of the exchange coupling between carriers and magnetic ions^47^. As a magnetic field is applied, the carrier mobility increases due to the suppression of the spin-dependent scattering by the magnetic polarons, giving rise to the negative MR, which is proportional to the susceptibility (*χ*(*H*))^29^,^43^ of the sample. However, the spin-dependent scattering effect by the magnetic polarons decreases with increasing temperature^43^, which makes the positive MR from the spatial fluctuations of magnetic polarons gradually show up. Due to the strong *p*–*d* exchange coupling^6^ in Mn_*x*_Ge_1−*x*_, the magnetic polarons will cause a strongly localized valence band splitting into two spin subbands *ɛ*=±
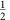
*SJM*(*r*)^43^,^48^, where *J* is the exchange coupling energy, *S* is the spin of electron and *M*(*r*) is the local magnetic moment of the magnetic polaron. This can form a complicated landscape for the valence band with hills and valleys, serving as the hole traps. With increasing the magnetic field, the hole traps become deeper, the number of itinerant holes decreases, and thus the MR increases. This positive MR is proportional to the square of the magnetization (*M*^2^(*H*))^29^, which can be reflected from the steep slope of the positive MR. As the temperature further increases, the contribution from the spin-dependent scattering becomes weaker, and finally only positive MR dominates the transport.

### Gate control of MR in the Mn_*x*_Ge_1−*x*_ nanomesh

**Figure 5:**
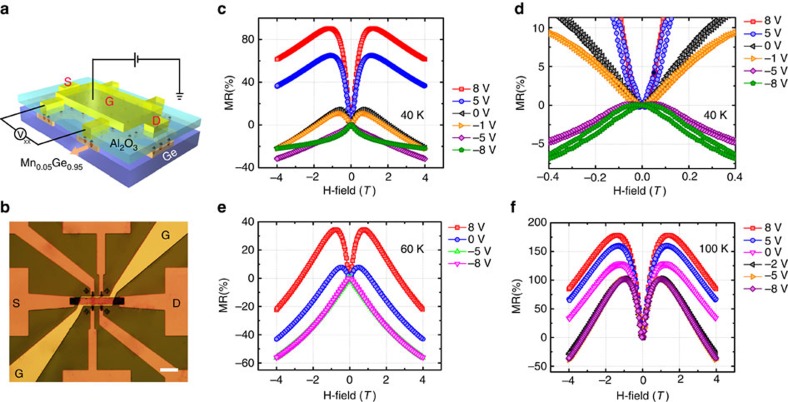
Electric-field-controlled magnetoresistance (MR) of Mn_x_Ge_1−x_ nanomeshes. (**a**) Schematic illustration and (**b**) Optical micrograph of the Mn_0.05_Ge_0.95_ nanomesh device for electric-field control of MR measurement, respectively. Scale bar, 50 μm. (**c**) Electric-field controlled MR at 40 K, clearly showing a transition from negative to positive when the gate was biased from negative to positive. (**d**) Magnified MR curves, clearly showing the biggest coercivity at −8 V. (**e**,**f**) Electric-field controlled MR transition at 60 and 100 K, respectively.

For low-power applications, it is desirable to exploit the electric-field control of ferromagnetism in our Mn_*x*_Ge_1−*x*_ nanomesh samples. For the measurement, an Al_2_O_3_ layer was deposited by atomic layer deposition on the nanomesh surface as the gate dielectric, followed by the e-beam evaporation of Cr/Au as the gate metal contact. Due to the unique nanomesh structure, the almost-wrap-around gate can be realized to provide a three-dimensional electric-field control of the conduction channel, thereby giving a very efficient and robust carrier modulation. The schematic illustration and optical micrograph of the gated nanomesh device are shown in Fig. 5a,b, respectively. Figure 5c–f shows the gate bias-dependent MR of the Mn_0.05_Ge_0.95_ nanomesh sample measured at 40, 60 and 100 K, while the comparison results on the Mn_0.03_Ge_0.97_ nanomesh sample are shown in Supplementary Fig. 4. When the gate bias changes from −8 to 8 V, a clear negative-to-positive MR transition is observed at 40 K. As described above, this phenomenon should also stem from the competitive effect between the spin-dependent scattering-induced negative MR and the spatial fluctuations-induced positive MR. As already reported, hole-mediated ferromagnetism in Mn-doped Ge^6^ has the ability to control the magnetic phase transition from a strong ferromagnetism to a soft ferromagnetism, when changing the gate bias from negative to positive. Therefore, as a negative gate bias is applied on our sample, the enhanced ferromagnetism with larger susceptibility (*χ*(*H*)) in the Mn_0.05_Ge_0.95_ nanomesh can provide a strong spin-dependent scattering, consequently giving rise to a negative MR. When the gate bias is swept to positive, the weakened ferromagnetism gives rise to a weaker spin-dependent scattering. From another point of view, the decreased density of magnetic polarons due to reduced carrier density probably makes them from an overlap-together state to a disconnecting state, which thus increases the polaron fluctuation, as the gate bias goes to the positive range. And hence the positive MR dominates. Additionally, from the zoom-in MR curves (shown in Fig. 5d), the biggest coercivity happens at −8 V with the highest hole density, which further confirms the hole-mediated ferromagnetism in our Mn_0.05_Ge_0.95_ nanomesh. To further illustrate the hole-mediated ferromagnetism, gate-dependent Hall measurements were performed on the Mn_0.05_Ge_0.95_ nanomesh, and the results are shown in Supplementary Fig. 5a,b. From the gate-dependent anomalous Hall term (*R*_xyA_) over longitudinal resistance (*R*_xx_), a hole-mediated ferromagnetism can be clearly observed, (see the details in Supplementary Note 3).

In addition, gate-dependent MR curves at 60 and 100 K are shown in Fig. 5e,f, respectively. At 60 K, the MR transition from the negative to positive value can still be clearly observed when tuning the gate bias. However, the control effect is not as strong as that at 40 K in the negative bias range. This effect can be explained by the fact that even at 0 V, the hole density at 60 K is already high enough to align most of the Mn ions along one direction^11^. Further increasing the negative bias does not significantly enhance the ferromagnetism. At 100 K, it shows a similar effect, that is, an even weaker control effect in the negative bias range. The gate-dependent Hall measurement at 100 K is shown in Supplementary Fig. 5c,d, which again shows the ferromagnetism modulation by the gate bias. Therefore, it is shown that by manipulating the Mn-doping concentration, not only giant MR but also electric-field controlled MR can be engineered. This property may offer a great advantage for designing future spintronics devices with voltage controlled, low-power and non-volatile functions.

## Discussion

To summarize, wafer-scale Mn_*x*_Ge_1−*x*_ nanomeshes with controlled Mn-doping concentration were successfully grown by MBE on a pre-patterned Ge substrate fabricated by nanosphere lithography. Comprehensive TEM characterization revealed its single crystallinity without observable intermetallic compounds formation. Extensive magnetic property measurements disclosed that the nanomesh exhibited a *T*_c_ over 400 K. Through magnetotransport measurement, two intriguing phenomena were found in such materials when adjusting the Mn-doping concentration: one was that the nanomesh exhibited a giant MR even up to 8,000%, and the other was that the MR could be engineered by external electric field. Both these features were attributed to the unique meshed nanostructures. The discovery of the extraordinary property in Mn_*x*_Ge_1−*x*_ nanomeshes points out an effective approach to synthesize high-quality and high-*T*_c_ FMS material. It may lay the foundation to build room-temperature spintronics devices, such as spinFET^9^, magnetic random-access memory^27^ and magnetic-field sensor^49^.

## Methods

### MBE growth

A ∼100 nm-thick SiO_2_ thin film was firstly deposited on a Ge (111) wafer by plasma enhanced chemical vapour deposition, followed by the formation of a large-scale and closely-packed hexagonal single layer of nanospheres on the SiO_2_ substrate (Fig. 1a). By adjusting O_2_ plasma etching time, the nanospheres were successfully shrunk to the desired size with appropriate gaps between the nanospheres as shown in Fig. 1b. Using the nanospheres as the mask, the pattern was transferred to the bottom SiO_2_ layer by two-step etching. The dry etching was firstly employed to etch SiO_2_ layer and stopped with ∼10 nm SiO_2_ left. Then, wet etching was hired to remove the left SiO_2_ layer. After dissolving the nanospheres, periodic SiO_2_ nanopillars were obtained on the substrate (shown in Fig. 1c). For the Mn_*x*_Ge_1−*x*_ nanomesh growth, the SiO_2_ nanopillars pattern was cleaned by O_2_ plasma to remove any organic residue induced by dry etching. Followed by rinsing in acetone, isopropyl alcohol and diluted hydrofluoric acid, the substrate was loaded into the MBE chamber for the Mn_*x*_Ge_1−*x*_ growth. High-purity Ge and Mn sources were evaporated from Knudsen effusion cells in MBE growth. After degassing at 600 °C for 30 min, the patterned substrate was *in-situ* cooled down to 160 °C for the Mn_*x*_Ge_1−*x*_ nanomesh growth with a Ge growth rate of ∼0.2 Å s^−1^ and a controlled Mn flux as the dopant source.

### Characterization

The morphologies of the patterns and formed nanomeshes were investigated by SEM. The microstructure and composition were comprehensively characterized by TEM equipped with EDS. SQUID was employed to measure their magnetic properties. Furthermore, the magnetotransport studies were carried out in the Quantum Design PPMS.

### Data availability

The data that support the findings of this study are available from the corresponding author upon request.

## Additional information

**How to cite this article:** Nie, T. *et al*. Enhancing electric-field control of ferromagnetism through nanoscale engineering of high-*T*_c_ Mn_*x*_Ge_1−*x*_ nanomesh. *Nat. Commun.* 7:12866 doi: 10.1038/ncomms12866 (2016).

## Supplementary Material

Supplementary Information Supplementary Figures 1-5, Supplementary Notes 1-3 and Supplementary References
