# Supplementary material for: Enhancing electric-field control of ferromagnetism through nanoscale engineering of high-Tc MnxGe1−x nanomesh
Source: Nat Commun. 2016 Oct 20;7:12866. doi: 10.1038/ncomms12866 (PMC5080415; doi:10.1038/ncomms12866)
Supplement: Supplementary Information — Supplementary Figures 1-5, Supplementary Notes 1-3 and Supplementary References [file ncomms12866-s1.pdf]

Supplementary Figures

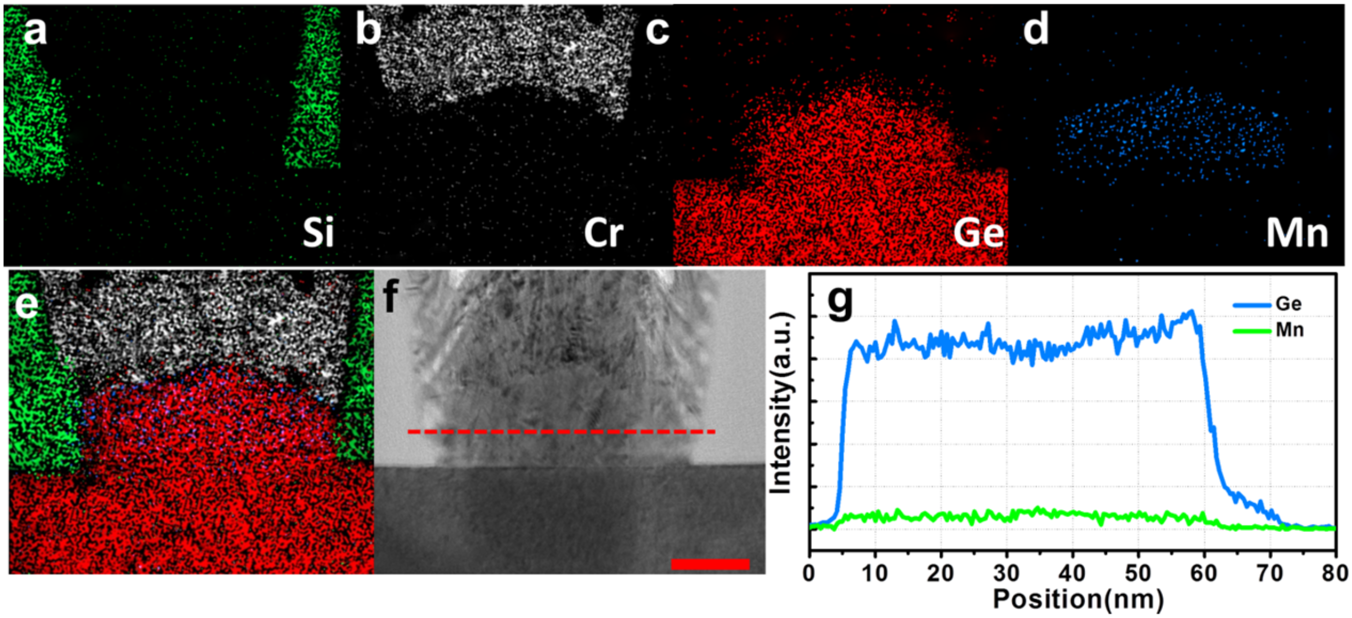

Supplementary Figure 1. | EDS mapping and line scan of the $Mn_{0.03}Ge_{0.97}$ nanomesh. a-d, EDS mapping from elements of Si, Cr, Ge and Mn, respectively, showing homogenous Mn distribution. **e,** EDS mapping of all elements. **f,** TEM image of the nanomesh. Scale bar, 20 nm. **g,** Line-scan EDS of Mn and Ge in the nanomesh, clearly indicating that Mn content in the nanomesh is about 3% with a homogenous distribution along the cross section.

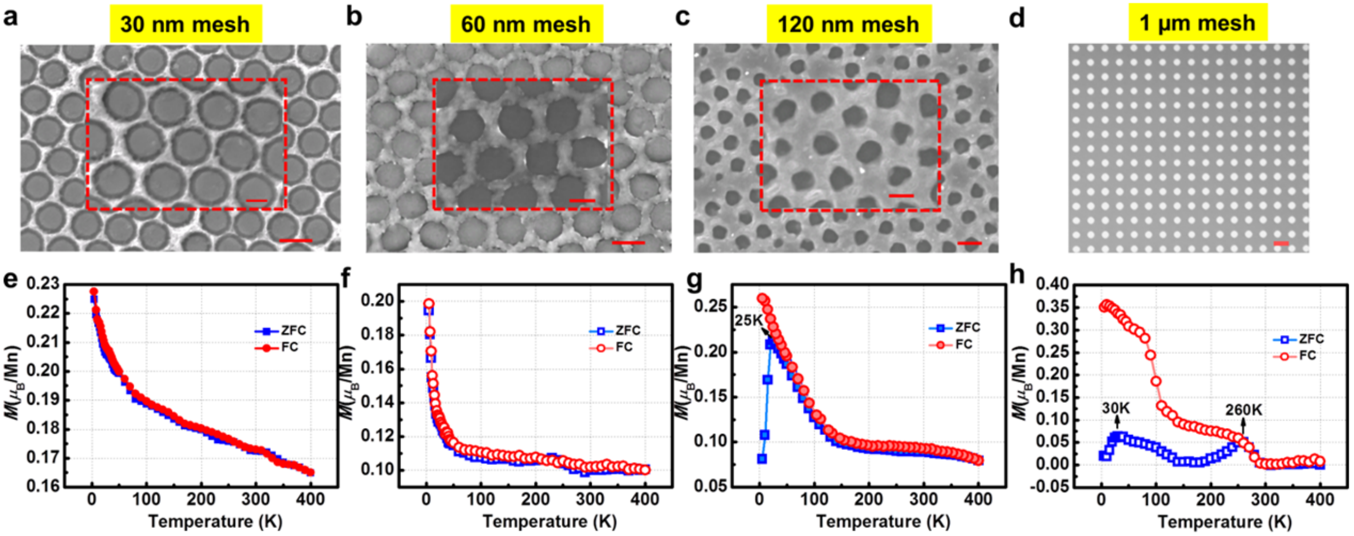

Supplementary Figure 2 | Size-dependent ZFC and FC curves in 5% Mn-doped mesh structure. **a-c,** Typical SEM images of the nanomeshes with width of 30 nm, 60 nm and 120 nm, respectively. Scale bar, 200 nm and 100 nm (in the insets). **d,** Optical micrograph of the μm-size mesh. Scale bar, 2 μm. **e-h,** Their corresponding ZFC and FC curves. The magnetic moment of all nanomesh samples still keeps a large value even at 400 K, indicating that the T_c of our nanomesh sample is above 400 K. The superimposed ZFC and FC curves without any blocking temperature indicate that the nanomeshes with 30 nm and 60 nm width can fully suppress the formation of Mn-rich nanophases and Mn_5Ge_3 precipitates. The blocking temperature at 25 K indicates the nanomesh with 120 nm width can form Mn-rich nanophases. However, the Mn_5Ge_3 precipitates can be suppressed. In the μm-size mesh, two blocking temperatures, i.e. 30 K and 260 K exist, which mean the μm-size mesh structure cannot suppress the formation of Mn-rich nanophases and Mn_5Ge_3 precipitates.

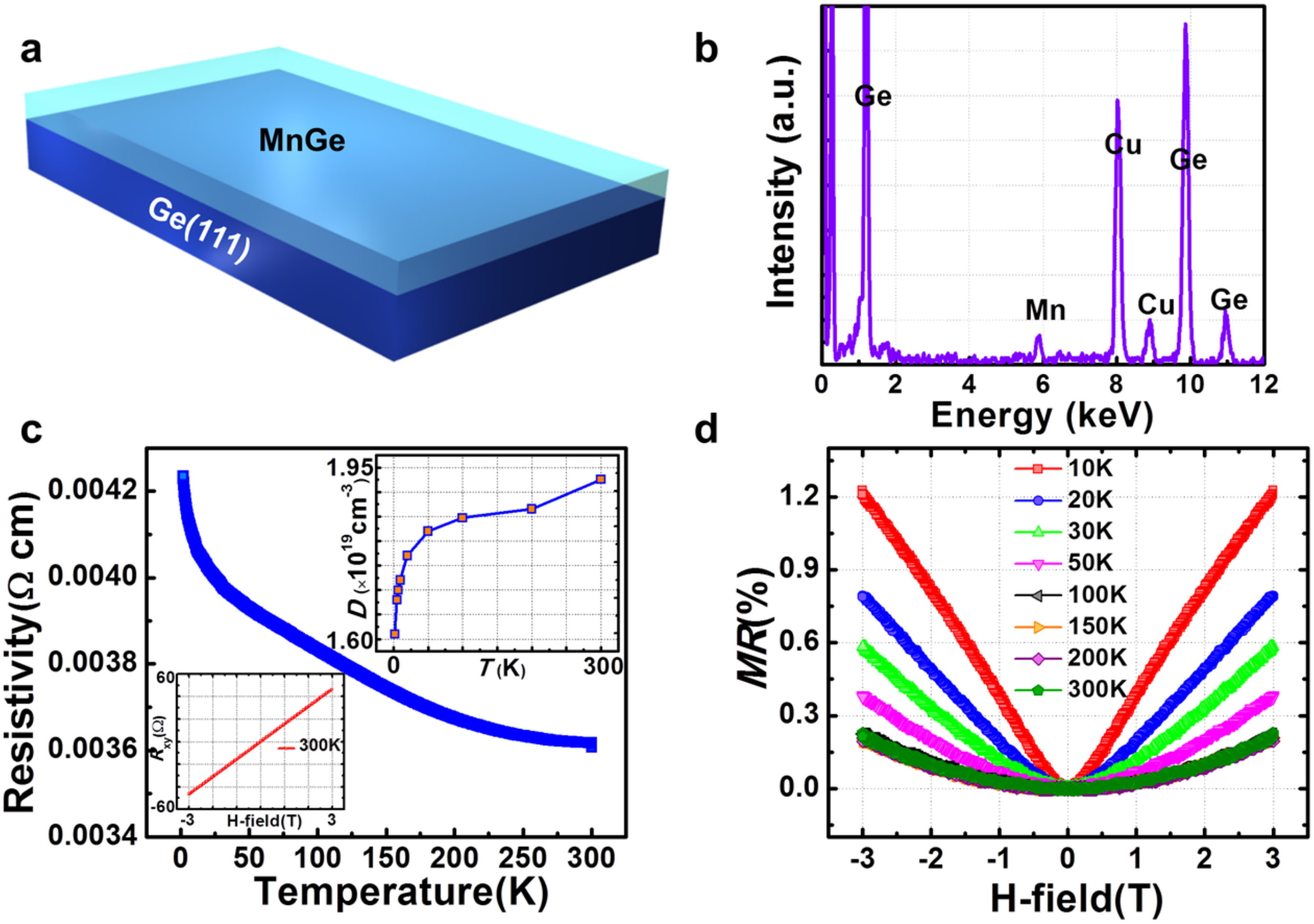

Supplementary Figure 3 | Properties of the Mn_xGe_{1-x} thin film. a, Schematic illustration of the Mn_xGe_{1-x} thin film. **b,** EDS spectrum of the Mn_xGe_{1-x} thin film, confirming the Mn doping concentration ~3%. **c,** Temperature-dependent resistivity of the Mn_xGe_{1-x} thin film. The top-right inset is the temperature-dependent carrier density. The bottom-left inset is the $R_{xy}(H)$ at 300 K. **d,** Temperature-dependent MR curves, showing a small positive MR in the entire temperature range.

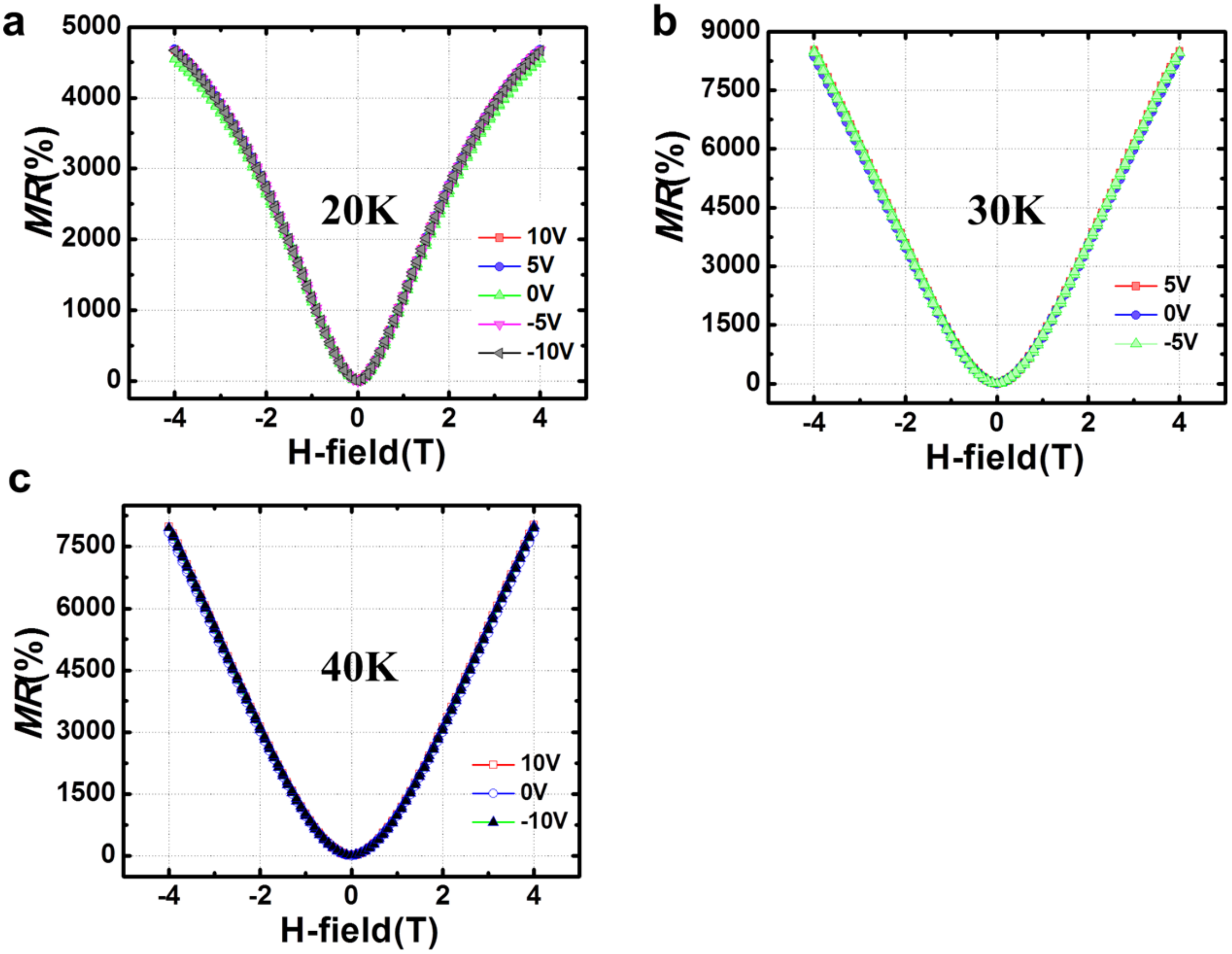

Supplementary Figure 4 | Magnetoresistance of the $Mn_{0.03}Ge_{0.97}$ nanomesh with the different gate bias. a, MR measurement at 20 K with sweeping the bias from -10 V to 10 V. An extremely large MR up to 5000% is clearly seen. There are no obvious changes of MR with gate bias. **b-c,** MR measurement with sweeping the bias at 30 K and 40 K, respectively. The MR in both of them shows no gate-bias dependence. As mentioned in the main text, the colossal MR comes from the geometrically enhanced MR and is not strongly related to the ferromagnetism state. Therefore, the almost gate-bias independent MR in the $Mn_{0.03}Ge_{0.97}$ nanomesh sample once again proves our explanation about the giant MR in the nanomesh structure.

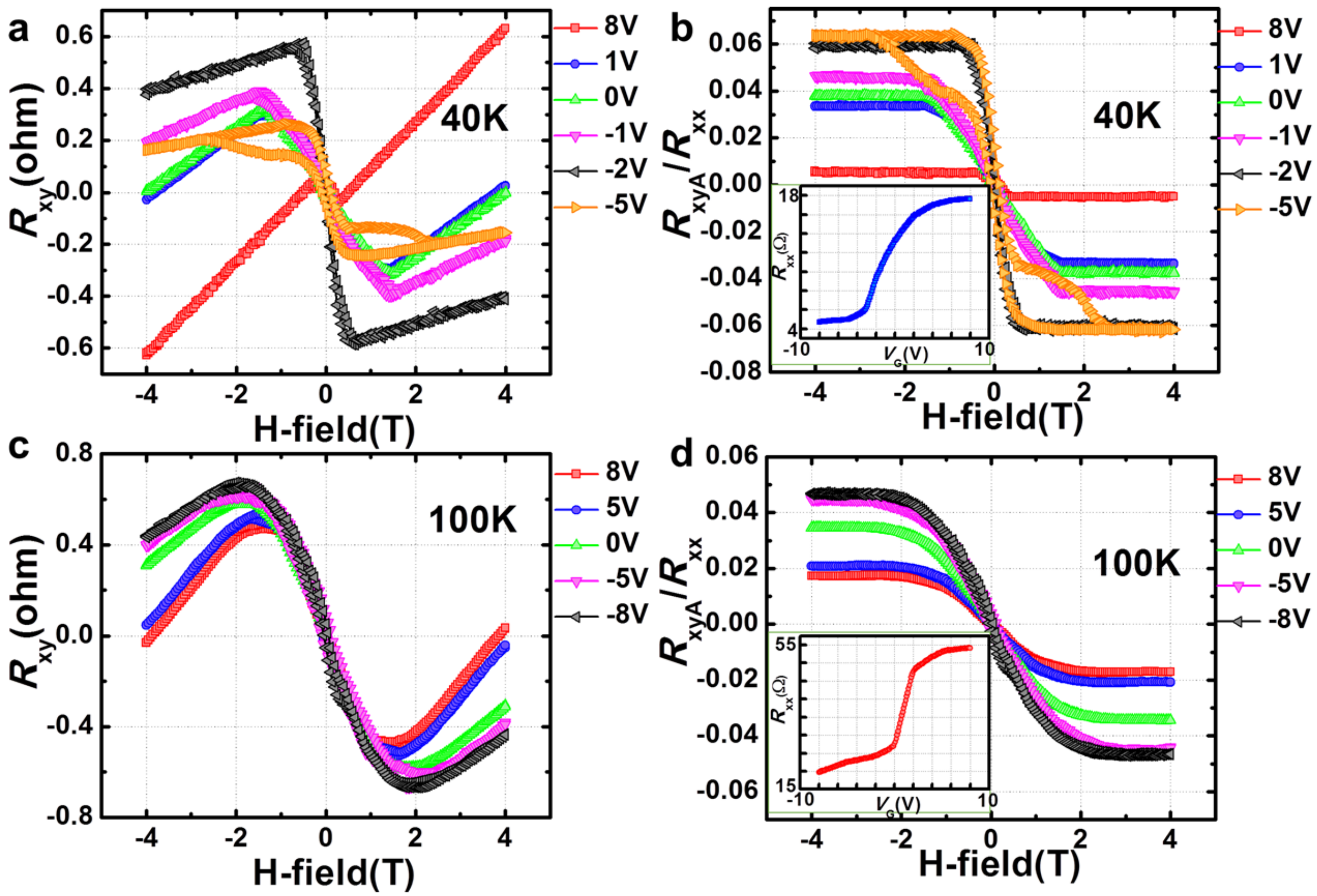

Supplementary Figure 5 | Gate-controlled anomalous Hall effect in the $Mn_{0.05}Ge_{0.95}$ nanomesh. a, Gate-dependent Hall resistance measured at 40 K, clearly showing the p-type characteristic of the $Mn_{0.05}Ge_{0.95}$ nanomesh. **b,** The anomalous Hall term (R_{xyA}) over R_{xx}, which is proportional to M, is plotted as a function of magnetic field, while sweeping the gate bias. The enhanced ferromagnetism in the negative bias, clearly confirms the hole-mediated ferromagnetism. The inset is the gate-dependent R_{xx}. **c,** Gate-dependent Hall resistance measured at 100 K. **d,** Gate-dependent R_{xyA}/R_{xx} versus magnetic field measured at 100 K. The inset is the gate-dependent R_{xx}, clearly demonstrating its p-type characteristic.

Supplementary Note 1 | Mesh size-dependent ZFC and FC curves

We give a systematic investigation of the size effect of the nanomesh on the magnetization. As mentioned previously, the nanospheres could be shrunk to the desired size by controlling O_2 plasma etching time. Then, we can get periodic SiO_2 nanopillar pattern with different diameter and gap. We can push the gap to the down limit of ~30 nm and up limit of ~120 nm. Based on these patterns, we grow nanomesh with the almost identical Mn-doping concentration of ~5 %. After removing the SiO_2 nanopillars, the formed $Mn_{0.05}Ge_{0.95}$ nanomeshes with width of ~30 nm, ~60 nm and ~120 nm were characterized by SEM and the typical morphologies are shown in Supplementary Fig. 2a-c, respectively. As a strong comparison, we also fabricated the μm-size pattern by photolithography for the μm-size mesh growth, and the optical micrograph is shown in Supplementary Fig. 2d. To understand the width-dependent magnetization and the possibly present nanophases[1], zero-field cooled (ZFC) and field cooled (FC) magnetization measurement were performed, and the results are shown in Supplementary Fig. 2e-h with the mesh width of 30 nm, 60 nm, 120 nm and 1 μm, respectively. The ZFC and FC data were recorded under an in-plane magnetic field of 100 Oe. There is a common feature that the magnetic moment of all nanomesh samples still keeps a large value even at 400 K, indicating that the T_c of our nanomesh sample is above 400 K, which confirms that nanomesh structure could significantly enhance the T_c. In comparison, the magnetization of the μm-size mesh has almost dropped down to zero at ~300 K. Further examination can find that the ZFC and FC curves are well superimposed with each other in the nanomeshes with 30 nm and 60 nm widths, indicating there are no any nanophase precipitates in both of them. And, we can further observe a slower decrease of magnetization with increasing temperature in 30 nm width nanomesh as compared to that in 60 nm width nanomesh, which indicates the ferromagnetic exchange interaction is enhanced in 30 nm width nanomesh. However, the nanomesh with 120 nm width shows a peak located at around 25 K in the ZFC curve. It has been well documented that two blocking temperature (T_b) usually could be observed in the Mn_xGe_{1-x} thin film with one at low

temperature and another one at high temperature[2]. The lower one was attributed to the presence of nanophase precipitates (Mn-rich Mn_xGe_{1-x} coherent nanostructures) resulting from spinodal decomposition[2, 3]. The higher one was interpreted as a superparamagnetic blocking phenomenon involving intermetallic precipitates (e.g. Mn_5Ge_3)[2, 3]. Therefore, we can conclude that the nanomesh with 120 nm width can form Mn-rich coherent Mn_xGe_{1-x} nanostructures. However, the Mn_5Ge_3 precipitates can be suppressed. In the μm-size mesh, both of the T_b exist, which means the μm-size mesh structure cannot suppress the formation of Mn-rich nanophases and Mn_5Ge_3 precipitates. The ~300 K T_c in μm-size mesh further proves existence of Mn_5Ge_3 precipitate, which has a T_c of 296 K[4]. We have also performed the ZFC and FC measurement on the thin film, which shows a similar feature (not present here) as that in the μm-size mesh. All of the results prove that the size/quantum confinement in Mn_xGe_{1-x} nanomesh structures can give a strong benefit to suppress the precipitates and improve the T_c.

Supplementary Note 2 | Mn_xGe_{1-x} thin film

For comparison, the Mn_xGe_{1-x} thin film (illustrated in Supplementary Fig. 3a) was grown simultaneously with the Mn_xGe_{1-x} nanomesh on an unpatterned Ge substrate under exactly the same growth condition. EDS can confirm that the Mn-doping concentration is ~3% (shown in Supplementary Fig. 3b), which is almost identical to that in the Mn_xGe_{1-x} nanomesh. The magnetotransport property of the Mn_xGe_{1-x} thin film was also measured by PPMS, and the results are displayed in Supplementary Fig. 3c-d. The temperature-dependent resistivity shows a semiconducting behavior, which is quite distinct from the case in the Mn_xGe_{1-x} nanomesh. A previous report[5] pointed out that the lattice strain associated with the substitutional Mn atoms can limit and control the growth of high-quality FMS. Above a certain critical strain, the Mn atoms would be segregated to form Mn-rich nanocrystals, occupying the interstitial positions of the Ge lattice[5]. Since Mn in the interstitial positions acts as donors, it compensates a significant fraction of itinerant holes generated from substitutional Mn, thereby reducing the effective carrier density[6]. The inset in Supplementary Fig. 3c clearly demonstrates the carrier density in thin film is in the range of 10^{19} cm^{-3}, which is much lower than that in nanomesh (close to 10^{21} cm^{-3}). Overall, the different transport behaviors between the Mn_xGe_{1-x} thin film and nanomesh suggest that Mn mainly occupies the substitutional site in the Mn_xGe_{1-x} nanomesh, which in turn contributes to the high T_c. Additionally, the temperature-dependent MR curves of the Mn_xGe_{1-x} thin film (Supplementary Fig. 3d) show only the positive MR in the whole temperature range with the maximum value of 1.2 % at 10 K under 3 T, much lower than the value of 8000% in the Mn_xGe_{1-x} nanomesh. The positive MR in the Mn_xGe_{1-x} thin film follows the parabolic dependence on the external magnetic field, suggesting that it comes from orbital MR effect[7]. Overall, the apparent deviation of the MR in the Mn_xGe_{1-x} thin film from that in the nanomesh indicates that the unique mesh structure indeed contributes to the giant MR, arising from a geometrically enhanced MR effect.

Supplementary Note 3 | Gate-controlled anomalous Hall effect in the $Mn_{0.05}Ge_{0.95}$ nanomesh

To further illustrate the electric-field controlled ferromagnetism in the $Mn_{0.05}Ge_{0.95}$ nanomesh, the gate-dependent anomalous Hall effect (AHE) was measured and the result is shown in Supplementary Fig. 5. The Hall resistance (R_{xy}) can be expressed as follows[8]:

$$R_{xy} = \frac{R_o}{d} B + \frac{R_A}{d} M \qquad (1)$$

where R_o is the ordinary Hall coefficient inversely proportional to the carrier density, R_A is the anomalous Hall coefficient[9], d is the sample thickness, and M is the magnetization of the sample. Thanks to the different origins of the asymmetric scattering of the spin polarized charge carriers in the presence of spin-orbit coupling, the anomalous Hall coefficient can show a linear or quadratic correlation with the longitudinal resistance, depicted as: $R_A = aR_{xx} + bR_{xx}^2$, where R_{xx} is the longitudinal resistance. In the Mn-doped Ge, the skew scattering mechanism usually dominates the anomalous Hall effect, giving rise to a linear dependence of R_A on R_{xx}[10, 11]. Supplementary Figure 5a shows the gate-dependent Hall resistance of the $Mn_{0.05}Ge_{0.95}$ nanomesh measured at 40 K. From the linear ordinary Hall term, the positive slope at 0 V indicates the $Mn_{0.05}Ge_{0.95}$ nanomesh is p-type, which further confirms most of the Mn as an acceptor in the substitutional site. More importantly, such slope dramatically decreases as the gate bias swept from 8 V to -8 V, indicating a well gate-modulation effect of the carriers from a hole depletion state (8 V) to a hole accumulation state (-8 V). To precisely elucidate the ferromagnetism of the sample, the linear ordinary Hall term was subtracted from the Hall resistance and the remained anomalous Hall term (R_{xyA}) over R_{xx} was plotted as a function of magnetic field, as shown in Supplementary Fig. 5b. It can be clearly seen that the smallest R_{xyA}/R_{xx} value happens at 8 V. As the gate bias is swept from positive to negative, the R_{xyA}/R_{xx} value dramatically increases. This obvious and robust magnetic phase transition that is from a weak ferromagnetism to a strong ferromagnetism as the bias from a positive to negative value, clearly demonstrates the hole-mediated

ferromagnetism in our samples. Supplementary Figure 5c is the gate-dependent Hall resistance measured at 100 K. Both the ordinary Hall term and the anomalous Hall term can be manipulated by the gate bias. For precisely demonstrating the gate control of ferromagnetism, the R_{xyA}/R_{xx} versus magnetic field was plotted in Supplementary Fig. 5d. It can be clearly seen that R_{xyA}/R_{xx} proportional to M, increases as the gate bias goes into the negative range, whereas the changes in the negative bias range are not so obvious, which may be due to the fact that the carrier density is already high enough to well align most of the Mn magnetization even at 0 V. Further increasing the carrier density cannot significantly enhance the ferromagnetism. The result agrees well with the gate-bias controlled MR at 100 K. In our sample, there exists a reverse sign of R_A in the $Mn_{0.05}Ge_{0.95}$ nanomesh. Previous reports[12,13] have pointed out that the sign of R_A was closely related to the logarithmic derivative of density of state $\left(\frac{\mathrm{d}(\ln N)}{\mathrm{d}E}\right)$ of the Fermion level, which could be changed by altering the hole trap, such as Mn interstitial[12], and thus was directly dependent on the growth condition.

Supplementary References

1. Nie T., *et al.* Superlattice of Fe_xGe_{1-x} nanodots and nanolayers for spintronics application. *Nanotechnology* **25**, 505702-505708 (2014).
2. Devillers T., *et al.* Structure and magnetism of self-organized Ge_{1-x} Mn_x nanocolumns on Ge (001). *Phys. Rev. B* **76**, 205306-205315 (2007).
3. Özer M. M., Thompson J. R. & Weitering H. H. Growth and magnetic properties of Mn-doped germanium near the kinetic solubility limit. *Phys. Rev. B* **85**, 125208-125214 (2012).
4. Jamet M., *et al.* High-Curie-temperature ferromagnetism in self-organized $Ge_{1-x}Mn_x$ nanocolumns. *Nat. Mater.* **5**, 653-659 (2006).
5. De Padova P., *et al.* $Mn_{0.06}Ge_{0.94}$ diluted magnetic semiconductor epitaxially grown on Ge(001): Influence of Mn_5Ge_3 nanoscopic clusters on the electronic and magnetic properties. *Phys. Rev. B* **77**, 045203-045209 (2008).
6. Li A. P., *et al.* Dopant segregation and giant magnetoresistance in manganese-doped germanium. *Phys. Rev. B* **75**, 201201-201204 (2007).
7. Park Y. D., *et al.* Magnetoresistance of Mn:Ge ferromagnetic nanoclusters in a diluted magnetic semiconductor matrix. *Appl. Phys. Lett.* **78**, 2739-2741 (2001).
8. Ohno H. Making Nonmagnetic Semiconductors Ferromagnetic. *Science* **281**, 951-956 (1998).
9. Nagaosa N., Sinova J., Onoda S., MacDonald A. & Ong N. Anomalous hall effect. *Rev. Mod. Phys.* **82**, 1539-1592 (2010).
10. Tsui F., *et al.* Novel Germanium-Based Magnetic Semiconductors. *Phys. Rev. Lett.* **91**, 177203-177206 (2003).
11. Li A. P., Wendelken J. F., Shen J., Feldman L. C., Thompson J. R. & Weitering H. H. Magnetism in Mn_xGe_{1-x} semiconductors mediated by impurity band carriers. *Phys. Rev. B* **72**, 195205-195213 (2005).
12. Allen W., Gwinn E. G., Kreutz T. C. & Gossard A. C. Anomalous Hall effect in ferromagnetic semiconductors with hopping transport. *Phys. Rev. B* **70**, 125320-125324 (2004).
13. Mi W. B., Liu Y. W., Jiang E. Y. & Bai H. L. Enhanced Hall effect in Fe_xGe_{1-x} nanocomposite films. *J. Appl. Phys.* **103**, 093713-093721 (2008).
